# Infrared Studies of Aragonite, Calcite, and Vaterite Type Structures in the Borates, Carbonates, and Nitrates

**DOI:** 10.6028/jres.065A.021

**Published:** 1961-06-01

**Authors:** C. E. Weir, Ellis R. Lippincott

## Abstract

Infrared absorption spectra have been obtained on the alkali nitrates, the divalent metal carbonates, and the rare earth borates which assume the aragonite, calcite, or vaterite crystal structures. It was observed that similar structures give rise to analogous spectra except for the carbonate and borates having the vaterite structure. The marked differences observed in these latter spectra are discussed. Frequency shifts produced by cation substitution are ascribed to repulsion between closed electron shells of oxygen atoms. It is concluded that this repulsive force determines the structure type in the rare earth borates.

## 1. Introduction

Although a considerable amount of work has been devoted to the study of the infrared spectra of calcite and aragonite type structures [1,2],^1^ relatively little attention has been given to vaterite [3]. The scarcity of the data on vaterite type structures is apparently due to the relative rarity of this structure. However, a large number of compounds with the vaterite-type structure have been prepared recently by Levin and Roth [4] in their studies of the rare-earth borates. They found that all rare-earth borates from SmBO_3_ to YbBO_3_ inclusive normally had the vaterite-type structure. Larger cations, such as Nd^3+^ formed borates with the aragonite-type structure and only the smallest, Lu^3+^, normally formed a borate with the calcite structure.

The availability of the borate compounds suggested a detailed study of the infrared spectra of the calcite-aragonite-vaterite type structures using a diamond infrared cell. This cell has the advantage that spectra are obtained routinely with no concern about interaction between the material studied and the diamond. Although some of the experiments on the calcite and aragonite spectra were performed with thin single crystals, most were run using pellet and mull techniques. In the latter methods there is uncertainty as to the effect on the spectrum of interaction between the pellet or mull material and the substance being studied.

The present report contains data on the spectra of calcite, aragonite, and vaterite type structures in the borate, carbonate, and nitrate series which were available. The data are analyzed to correlate the spectra with structure in order to obtain information on the interatomic forces and the reasons for the occurrence of the different structure types in the rare earth borates. Representative data for a few other structures of interest are also included.

## 2. Apparatus and Experimental Method

All absorption spectra were obtained using a type-II diamond cell [1] in a commercially available infrared spectrometer. The region from 6*μ* to 20*μ* was covered in these experiments.

In a given experiment a few milligrams of powder or a small crystal of the specimen was placed on one diamond surface. The cell was assembled and a maximum pressure of a few hundred atmospheres was applied to produce a clear film. The pressure was decreased to a few atmospheres, e.g., less than 50 atm, before obtaining the spectra. Since the specimen was in contact with diamond alone no shift in bands from interaction with the diamond is to be expected. Previous studies have shown that the positions of the bands are essentially unaffected by the low pressures used in these measurements.

All nitrates studied were reagent grade chemicals. All carbonates except vaterite were of natural origin with the small specimens required being obtained from small single crystals. The structure type was verified by X-rays in those instances where there was any uncertainty. Most of the natural minerals have been in use here for several years as optical and X-ray standards. Vaterite^2^ was prepared according to the procedure described by Wray and Daniels [5] and the structure was confirmed by X-ray analysis. The X-ray pattern indicated the presence of a small amount of calcite and this was confirmed microscopically. The borate samples were prepared by Levin and Roth [4] from the rare-earth oxides and orthoboric acid by ordinary solid state reaction techniques. A contaminant consisting of a 3R_2_O_3_·B_2_O_3_ high temperature phase may have been present in some of the borates.

## 3. Infrared Spectral Analysis

For the isolated planar ion XO_3_ having trigonal symmetry there are four fundamental modes of vibration; the symmetric stretching, *v*_1_, the out of plane bending, *v*_2_, the doubly degenerate antisymmetric stretching, *v*_3_, and the doubly degenerate planar bending, *v*_4_ [6]. Of these fundamentals, three are inherently active in the infrared while the fourth, the symmetric stretching, is inactive in isolated ions. In a crystalline solid containing more than 1 moleculeper unit cell, symmetry considerations indicate that all modes may be active and coupling between various modes may even remove degeneracies [7, 8]. In the calcite structure it is expected that *v*_2_,* v*_3_, and *v*_4_ will be observed with *v*_1_ inactive and both *v*_3_ and *v*_4_ doubly degenerate. In aragonite, six bands are expected since in this structure *v*_1_ is active and the degeneracies are removed from *v*_3_ and *v*_4_. Vaterite is reported to have a hexagonal cell containing two or more molecules per unit cell [9, 10, 11]. However, the structural details of the unit cell have not been established so far as can be ascertained. Therefore, the expected spectrum cannot be predicted.

The errors associated with measuring the frequencies may be assessed as follows: All values recorded represent average peak positions. In general, *v*_1_ (not usually observed in calcite), is very sharp and its position can be determined with little error. Both *v*_2_ and *v*_4_ are reasonably sharp and occur in a region of high dispersion. These values are also considered to be known with little error. In all specimens, *v*_3_ is extremely strong and broad and is distinctly asymmetrical. The broadness coupled with the location of *v*_3_ in a region of low dispersion imply that the tabulated *v*_3_ values are subject to some uncertainty. In particular, it is to be noted that as a result of asymmetry, *v*_3_ values corresponding to positions of maximum absorption will differ considerably from the values tabulated. Comparison of the data of this report with the data of Huang and Kerr [15] for carbonates shows that *v*_2_ and *v*_4_ values agree within ±2 cm^−1^ but that corresponding values of *v*_3_ may differ by as much as 40 cm^−1^.

## 4. Results

### 4.1. Calcite

**Figure 1:**
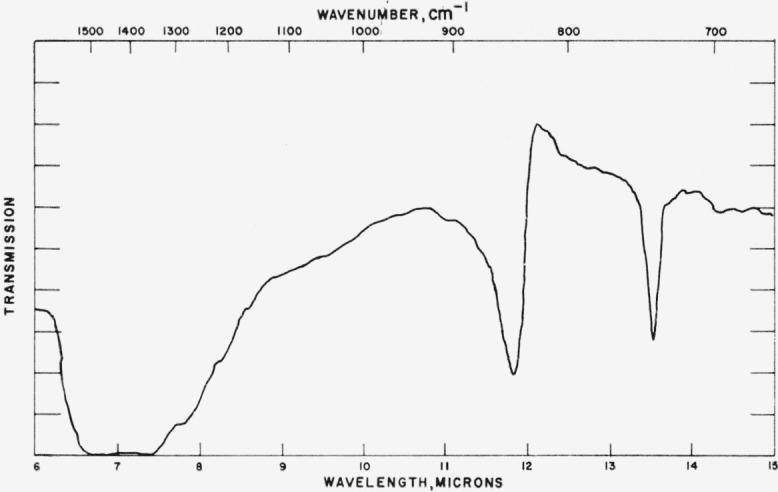
Infrared absorption spectrum of LuBO_3_ (calcite structure).

**Figure 2 f2-jresv65an3p173_a1b:**
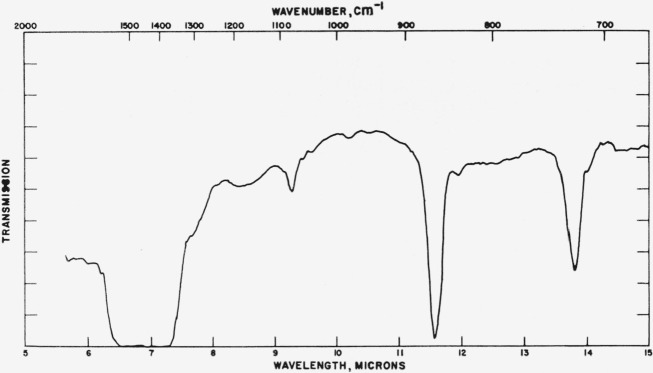
Infrared absorption spectrum of CdCO_3_ (calcite structure).

**Figure 3:**
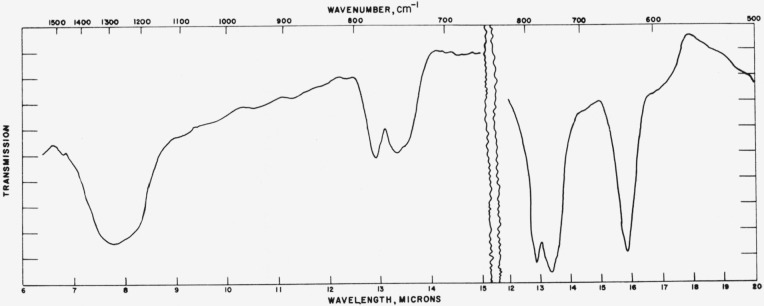
Infrared absorption spectrum of LuBO_2_ (calcite structure).

Typical spectra for the calcite structures are shown in figures 1 to 3 and the data for all calcite structures studied are given in table 1. The tabular data consist of the frequencies in cm^−1^, the force constants calculated from the observed frequencies assuming a simple valence force potential for the isolated XO_3_ ion [6], the ionic radius of the cation [11], and the unit cell constants [12, 13]. Values for *v*_1_, which is inactive in calcite type structures, are available for NaNO_3_ and CaCO_3_ from Raman spectra [14]. In CdCO_3_ and CoCO_3_ bands were observed in the position expected for *v*_1_ and these are listed in the table. All other values for *v*_1_ are given in parentheses and are assumed to be identical with the corresponding values found for NaNO_3_, CaCO_3_ or NdBO_3_ (aragonite type structure). From data to be given later, it appears that the error arising from the assumed values of *v*_1_ do not exceed a few percent. However, force constants involving the assumed values of *v*_1_ will reflect the error and such values are also enclosed in parentheses. The arrangement in table 1 is in the order of increasing valence of anion with subarrangement in the order of increasing ionic radius of cation. [Fig f2-jresv65an3p173_a1b]

In general, the results listed in table 1 are in agreement with the spectra predicted on the planar ion model. Several unexpected bands are found, however, i.e., weak low frequency satellites of the *v*_2_ bands in CdCO_3_ and CoCO_3_ and the appearance of the *v*_1_ band in these materials. There is also a reasonably strong high frequency satellite of *v*_2_ in the borates. This band has been shown by Steele and Decius [16] to arise from the B^10^ isotope. Decius [17] has discussed the splitting and coupling of the out of plane bending modes to be expected under these conditions. The low frequency satellite of *v*_2_ in the carbonates is probably not due to a similar isotope effect because of the rarity of C^14^. It would appear that the band might arise from coupling of out of plane modes of adjacent XO_3_ ions, but according to the treatment of Decius [17], electrostatic coupling is not to be expected in the vibrations unless mass differences exist in the XO_3_ ions. There is little doubt that a similar satellite of the *v*_2_ band occurs in most of the carbonates although it is of low intensity and is not listed in table 1. That coupling of modes between adjacent CO_3_^=^ ions may be the cause of the satellite is indicated by the fact that the distance between adjacent CO_3_^=^ ions along the *c* axis of CoCO_3_ is smaller than that in MgCO_3_; and is smaller in CdCO_3_ than in CaCO_3_. Whatever the origin of the satellite of *v*_2_, interaction effects appear to be responsible for the appearance of *v*_1_ in CdCO_3_ and CoCO_3_ since the band obviously contains fine structure, being much broader than observed in any other similar materials. The broad, asymmetric appearance of *v*_3_ is also indicative of coupling or interaction between the antisymmetric mode and other vibrations.

The data for dolomite are of particular interest inasmuch as alternate cation positions are occupied by Ca and Mg ions [13]. From the crystal structure it might be expected that the larger Ca ion would influence the out of plane and antisymmetric vibrations and that the average field of both ions would affect the in-plane vibration. It is found that in dolomite *v*_2_ agrees with that for calcite and *v*_4_ is approximately the average of the corresponding values for calcite and magnesite. However, contrary to expectations, *v*_3_ for dolomite is almost identical with *v*_3_ for magnesite.

The data of table 1 do not illustrate clearly the trend of frequency with ionic radius of cation. There is some indication that the in-plane frequency increases as the cation radius decreases. However, it is most likely that all the carbonates listed are not equally ionic and effects due to variation in the ionic character of the compound will probably be superimposed on effects arising from ionic size. In the alkaline earth carbonates and the alkali nitrates which may be considered to be completely ionic it appears from the tabulated frequencies that smaller cations produce frequency shifts to higher energies. This trend will become more apparent in data presented later.

### 4.2. Aragonite

**Figure 4:**
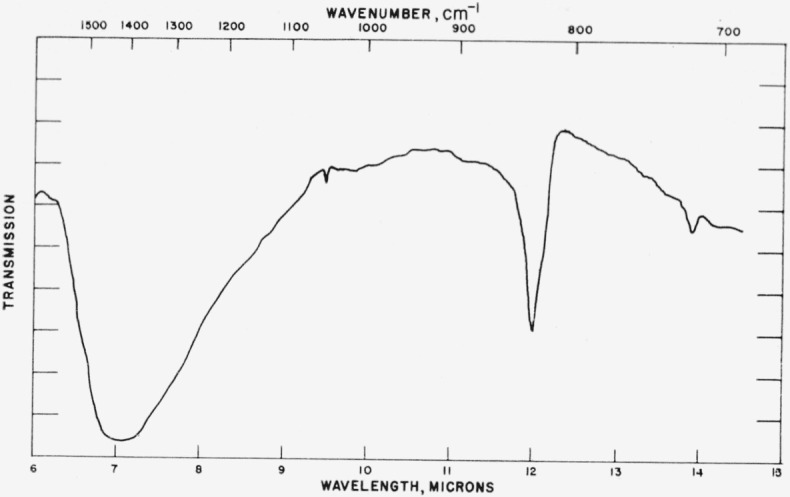
Infrared absorption spectrum of KNO_3_ (aragonite structure).

**Figure 5 f5-jresv65an3p173_a1b:**
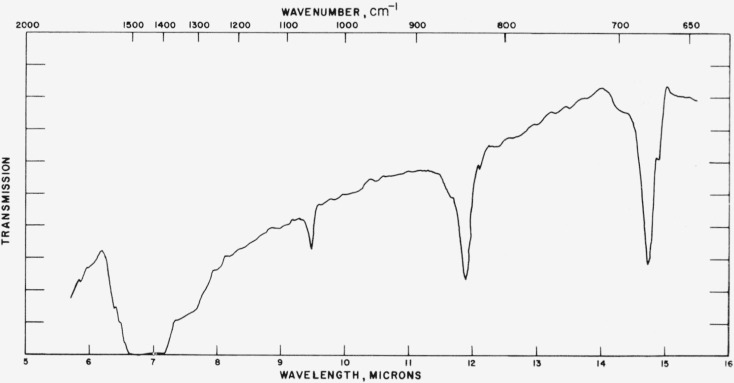
Infrared absorption spectrum of PbCO_3_ (aragonite structure).

**Figure 6:**
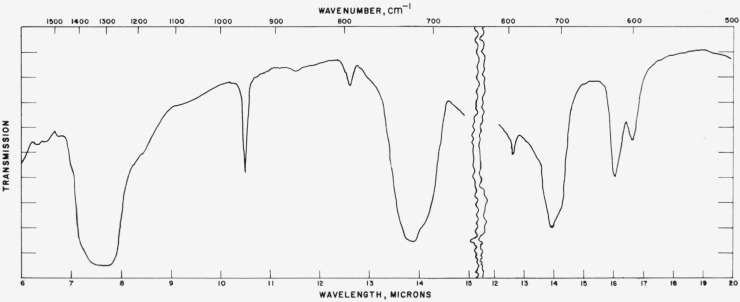
Infrared absorption spectrum of NdBO_3_ (aragonite structure).

Typical absorption spectra of aragonite type structures are shown in figures 4 to 6 and the pertinent data are compiled in table 2. The arrangement in table 2 follows that of table 1. The tabular data show that only for CaCO_3_ is there good evidence for the splitting of *v*_3_. For all other materials, however, it is apparent that the *v*_3_ band contains internal structure unresolved by the spectrometer which is consistent with the predicted splitting. The two components of *v*_4_ appear in all specimens except KNO_3_ in which *v*_4_ is very weak and apparently unsplit. It should be noted that in all cases where the *v*_4_ band is split into two components that the higher frequency component is invariably much the stronger. As in the calcite structure there is evidence for a low frequency component on most of the *v*_2_ bands in the carbonates. In the borates the second component of *v*_2_ is quite strong and most probably due to B^10^ [16]. The effect of change in ionic radius of cation and differences in packing in the unit cell is shown by the trends of *v*_1_ and *k* in the alkaline earth carbonates. The increase in *v*_1_ and *k* as the unit cell decreases in size may be attributed to shortening of the C—O bond by repulsion of closed shells of the oxygen atoms. It will be noted that PbCO_3_ does not follow the trend for the alkaline earth carbonates, a behavior which is probably due to the greater covalency of the Pb—O bonds. [Fig f5-jresv65an3p173_a1b]

Comparison of the force constants and frequencies for CaCO_3_ in the aragonite and calcite structures shows that there is little change. This is particularly true in the stretching force constants. A similar conclusion is reached by comparing data for NaNO_3_ and KNO_3_. Since the cation coordination number is six in calcite and nine in aragonite [18] it is concluded that the electrostatic forces around the ionic cations have little effect on the internal vibrations of the much more tightly bonded anions. It can also be concluded that changes in vibrational frequencies are primarily due to anion-anion forces rather than anion-cation interactions.

### 4.3. Vaterite

**Figure 7:**
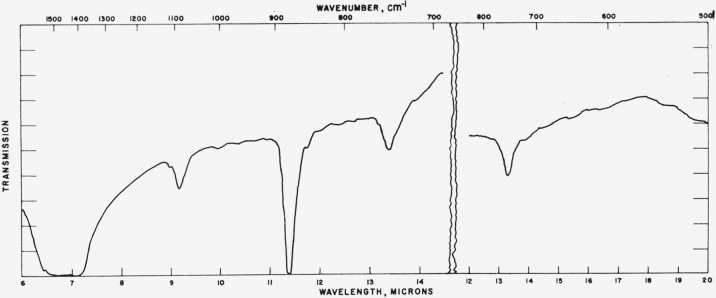
Infrared absorption spectrum of CaCO_3_ (vaterite structure).

**Figure 8 f8-jresv65an3p173_a1b:**
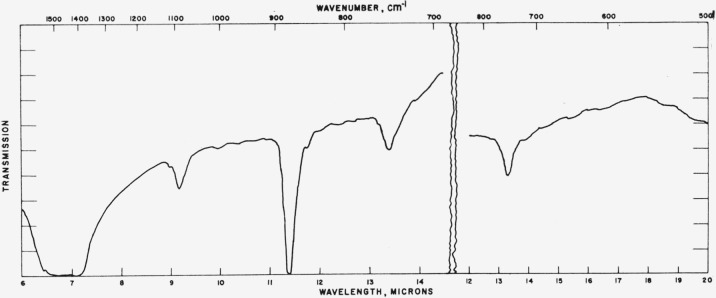
Infrared absorption spectrum of SmBO_3_ (vaterite structure).

**Figure 9 f9-jresv65an3p173_a1b:**
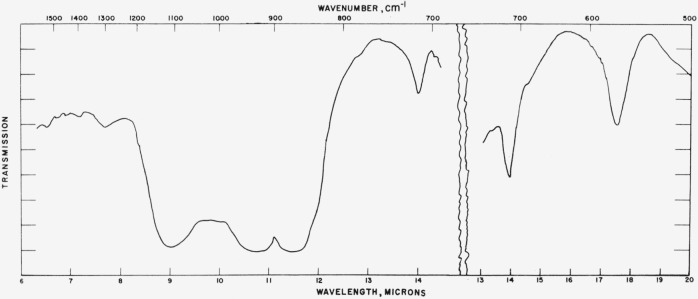
Infrared absorption spectrum of YBO_3_ (vaterite structure).

**Figure 10:**
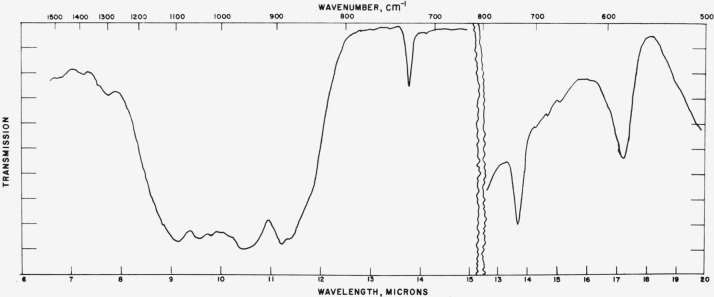
Infrared absorption spectrum of LuBO_3_ (vaterite structure).

Typical absorption spectra for the vaterite type structures are shown in figures 7 to 10 and the data are compiled in table 3. The spectrum of the vaterite form of CaCO_3_ is analogous to those for calcite and aragonite, and the frequency assignments appear straight forward. Accordingly, the complete tabular data are given for CaCO_3_. The spectra of the borate type vaterites are considerably different from those for the borates in the calcite and aragonite structures. The borate vaterites are characterized by an extremely broad and intense absorption band extending from 800 cm^−1^ to 1,200 cm^−1^. Studies made on extremely thin films showed that this one intense band consisted most probably of four broad bands. Of these four, the existence of three is unequivocal and the average positions of the bands could be located with reasonable accuracy. The fourth band, however, appeared to be definite for some specimens but doubtful for others. This band occurs near 1,000 cm^−1^, is weaker than the adjoining bands and is partially obscured. Inasmuch as it was not observed definitely in all samples and could not be located with reliability when it appeared, the data for the fourth band near 1,000 cm^−1^ are omitted from the table. The band assignments have not been listed in table 3 for the vaterite type borates. It seems likely that the bands below 800 cm^−1^ arise from distortion and bending modes and they are so tabulated. Bands above 800 cm^−1^ are designated as stretching modes. It is possible that the symmetric stretching band is that near 930 cm^−1^ in the vaterites in analogy with the value found in the borate type aragonites. However, this is by no means certain since the character of the band is quite different in the two instances, i.e., sharp and of medium intensity in aragonite, and broad and very intense in vaterite. [Fig f8-jresv65an3p173_a1b] [Fig f9-jresv65an3p173_a1b]

The analysis of the spectra on the borates was believed originally to be complicated by the existence of the 3R_2_O_3_ B_2_O_3_ compound and the high temperature forms of low symmetry reported by Levin and Roth [4]. Patterns containing high percentages of Tm_2_O_3_ were used to identify the spectrum of 3Tm_2_O_3_·B_2_O_3_. It was found that a broad, very strong band near 1,300 cm^−1^ was the most prominent feature in the spectrum of this material. Traces of this band were observed in most spectra of the borate type vaterites, but the other bands being weaker were not observed. The band near 1,300 cm^−1^ is not listed in table 3, as it is believed to arise from the 3:1 compound.

The infrared patterns of the carbonate and borate vaterite type structures are so strikingly dissimilar that it is difficult to believe that they arise from isostructural compounds. However, the X-ray data show that it would be equally difficult to conclude that the compounds are not isostructural [4]. The absorption pattern of the carbonate type vaterite agrees in detail with a spectrum published previously [3]. Comparison of the data for the carbonate type vaterite with aragonite and calcite shows that the stretching force constant and the out-of-plane bending constant are essentially unchanged. However, the in-plane constant has increased in vaterite. If the analysis of the effects of different types of forces in solution by Benson and Drickamer [19] is considered to apply here, the marked change in the in-plane bending constant is indicative of repulsion between oxygen atoms in the plane of the ion.

Ordinarily the borate type vaterite spectrum would be interpreted in terms of an increase in B—O distances as compared with the corresponding distances in the other polymorphic forms. This change would imply that an increase in coordination of boron had occurred in the transition from the calcite or aragonite to the vaterite type structure. The indication from the data of strong shifts of all bands to lower frequencies in the borate type vaterite structure supports these conclusions. However, the indication of such shifts is not necessarily correct as it is not possible to identify the modes of vibration. Boron is known to assume a tetrahedral fourfold coordination in many compounds and may exhibit both 3- and 4-fold coordination simultaneously in some materials [20, 21]. It is found that as the radius of the rare earth ion decreases, the structure of the rare earth borate changes from aragonite type to vaterite type, and then to calcite type. It seems very improbable that the coordination number of the boron should change from three to four and then revert to three again as the size of the cation decreases monotonically. Therefore, despite the indications of the infrared data, it appears unwise to ascribe the spectrum of the borate to boron in 4-fold coordination. Under the present circumstances it is possible to conclude only that borate and possibly carbonate ions are subjected to severe perturbations in the vaterite type structure. The dissimilarity of the infrared spectra and the similarity of the structures of the borates as shown by X-ray data raise interesting questions to be answered in future studies.

### 4.4. Miscellaneous Structures

**Figure 11:**
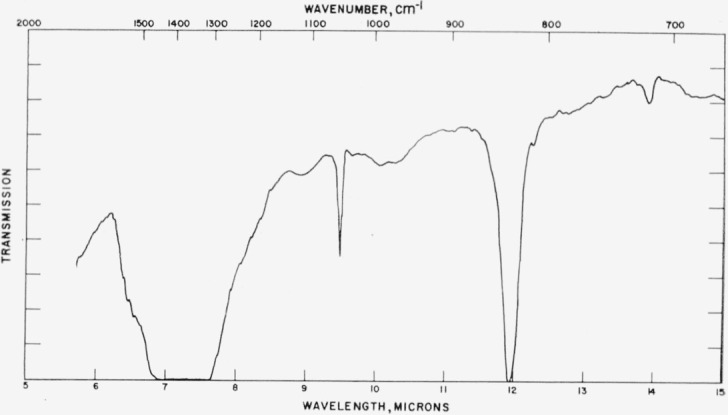
Infrared absorption spectrum of CsNO_3._

**Figure 12 f12-jresv65an3p173_a1b:**
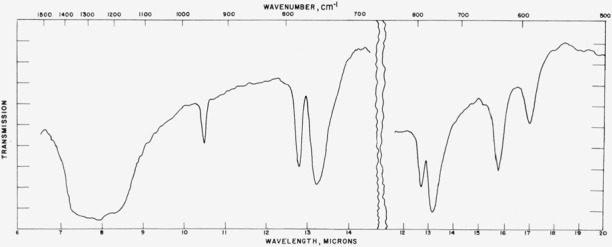
Infrared absorption spectrum of LaBO_3_ (high temperature structure).

**Figure 13:**
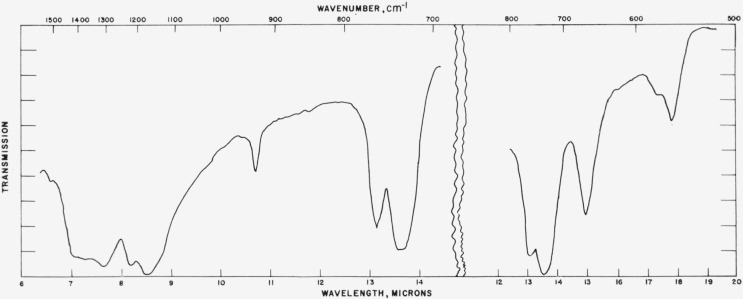
Infrared absorption spectrum of NdBO_3_ (high temperature structure).

Absorption spectra for other materials in this series are given in figures 11 to 13 and the data are compiled in table 4. For CsNO_3_ the data are arranged as in table 1. The high temperature borates exist metastably at room temperature and the spectra were obtained at room temperature. These materials are of unknown structure type of low symmetry and the spectral assignments are unknown. By analogy with other borates it would appear that the sharp band near 950 cm^−1^ represents the symmetric stretching mode, but further assignments are not obvious. In general, the complexity of the spectrum confirms the low symmetry found by X-ray analysis [4]. [Fig f12-jresv65an3p173_a1b]

## 5. Discussion

### 5.1. Effect of Mass of Cation

It has been suggested [15, 22] that change in the mass of the cation is reflected by a change in the observed internal frequencies of the anion through a modified inverse square root relationship. It would appear that the mass of the cation should have a small effect on the internal anion frequencies since the bond between the anion and cation for purely ionic bonding is much weaker than the internal covalent bonds of the anion. It was noted previously that in calcium carbonate a change in coordination number of the calcium ion in the transition from calcite to aragonite produces a negligible frequency shift. The data on the borate type vaterites refute the idea of a conventional mass effect. Although the mass of the cation increases from Sm^3+^ to Lu^3+^, it is apparent that the frequencies increase also from SmBO_3_ to LuBO_3_. Considering YBO_3_, in which the cation has a mass approximately half that of the rare earth ions, it is observed that the frequencies are not much higher as expected by conventional mass effects but close to what would be expected on the basis of the radius of the cation. Similarly, there is no correlation of frequency with mass of cation in the carbonate type calcites. There appear to be irregularities in the carbonates which are most readily interpreted as change in ionic character of the cation-oxygen bond such as in PbCO_3_. It seems quite reasonable to conclude that as this bond becomes more covalent the cation mass may play a greater role in affecting the internal vibrations of the anion.

### 5.2. Effect of Volume

The data on the rare earth borates of the vaterite structure type lead to other interesting conclusions. The rare earth ions form a series whose external electron configurations are quite similar. Quantitatively these ions should show very small differences in the character of their interactions with their neighbors in the crystal. In the vaterite structures, as the cation is changed from Sm^3+^ to Lu^3+^, the ionic radius decreases by some 15 percent. This result may be considered to be produced by an increase in the *effective* nuclear charge tending to reduce the size of the electron shells.

The unit cell dimensions also become smaller as the cation radius decreases and as a consequence the cation-oxygen nuclear distance becomes less. Both the increase in effective charge and the smaller cation-oxygen distance cause an increase in *electrostatic* attraction of cation for oxygen. The increased attractive force must be balanced by an increased repulsion that is most readily attributed to overlap of closed electron shells of the oxygen atoms which comprise the major portion of the unit cell volume. However, an increase in the oxygen-oxygen repulsion should be reflected in a corresponding shortening of the B—O bond and an increase in the frequencies of vibration of the borate ion. This trend is illustrated by the data on the borate type vaterites, the alkaline earth type aragonites, the nitrate type calcites, etc. Data on garnets with various cations [22] also show the same effect. It will be noted that the data for YBO_3_ do not conform exactly to the order of the rare earth borates. Since Y^3+^ has a different electronic configuration than the rare earth ions, this is not surprising. The differences may readily be attributed to the difference in the oxygen-oxygen interactions caused by the variation in the outer shell electrons of the Y^3+^ and the rare earth ions.

### 5.3. Effect of Ionic Radius on Structure Type

**Figure 14:**
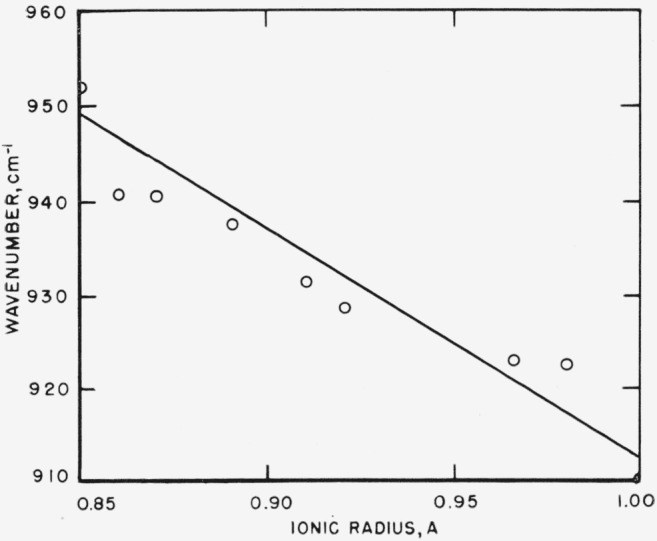
Effect of ionic radius of cation on position of 930 cm^−l^ band in borate type vaterites.

**Figure 15 f15-jresv65an3p173_a1b:**
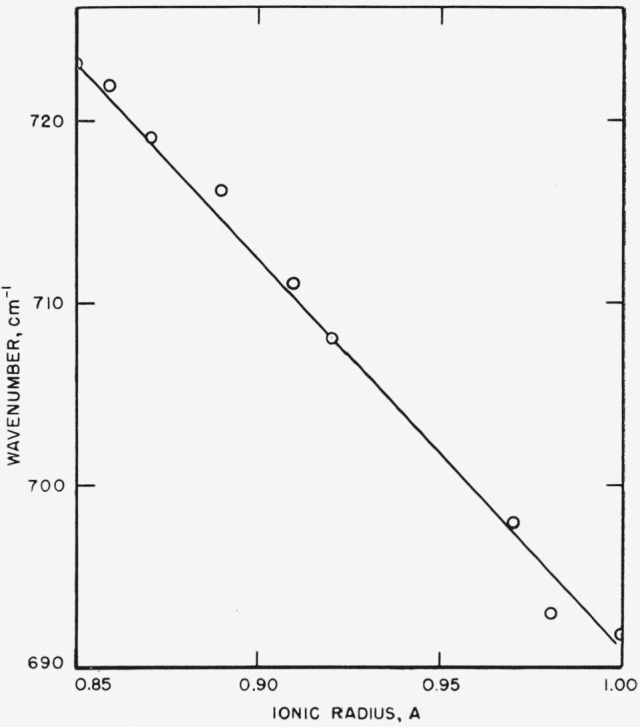
Effect of ionic radius of cation on position of 710 cm^−1^ band in borate type vaterites.

**Figure 16:**
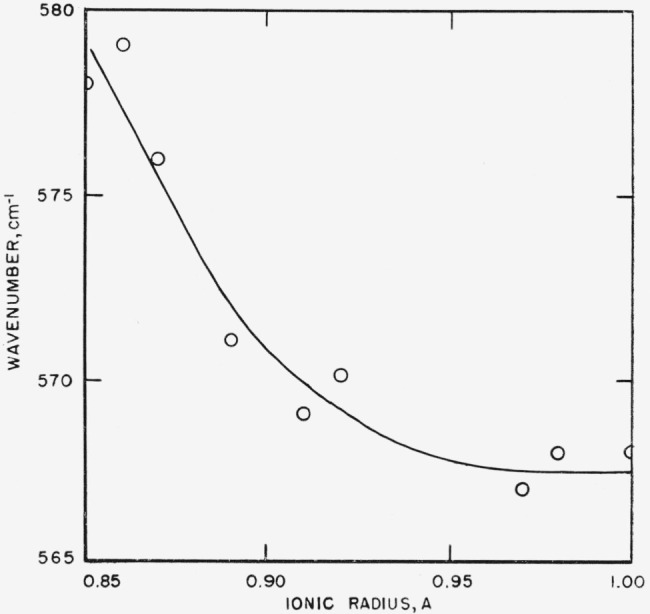
Effect of ionic radius of cation on position of 570 cm^−1^ band in borate type vaterites.

The present data shed some light on the causes of changes in structure produced by variation in the ionic radius. As noted, the frequency trend observed in these studies is readily interpreted as arising from repulsion of oxygen atoms in the crystals. Figures 14 to 16 show the frequency-ionic radius dependence for three bands in the borate type vaterites. For the 900 cm^−1^ and 700 cm^−1^ bands, the frequencies increase essentially linearly as the ionic radius diminishes. The 550 cm^−1^ band, however, shows a rapidly accelerating increase in frequency as the ionic radius decreases. From figure 16 it is apparent that the energy associated with this vibration is rising rapidly. It seems most reasonable to suppose that eventually a different arrangement of the structural units becomes energetically more favorable, and the new structure will be assumed if the energy barrier can be surmounted. Thus, as the cation is changed from Yb^3+^, for which vaterite is stable to the slightly smaller Lu^3+^, the vaterite structure is not stable because of the greater repulsive forces, and the more open calcite structure is formed. Conversely as the atomic number of the cation is decreased, the closed shell repulsive forces decrease very rapidly whereas the electrostatic attractive forces decrease at a slower rate, and the energy will rise along the attractive leg of the potential energy curve until a point is reached, at which another more dense structure (aragonite) becomes stable. This occurs between Sm^3+^ and Nd^3+^. That these ideas are qualitatively consistent is indicated by the fact that at elevated temperatures at which the O—O repulsive forces will decrease because of thermal expansion, the calcite form of LuBO_3_ reverts to the vaterite type structure which is more stable in the expanded lattice. [Fig f15-jresv65an3p173_a1b]

The considerations outlined apply only to structure type changes produced by ionic substitution. The question of a general polymorphic transition when produced by a change in pressure or temperature, or both, is considerably more complex inasmuch as kinetic as well as potential energies are involved. However, it appears that repulsive energy considerations discussed here may well be the governing factor in “abnormal” transitions in which the high temperature phase is denser than the low temperature phase.

## Figures and Tables

**Table 1:** Infrared frequencies and related data for calcite structures

Compound	Frequency, cm^−1^				Force constant,^1^ dyne/cm× 10^−5^			Ionic radius of cation,^2^ A	Unit cell constants,^3^ A	

	*v*_1_	*v*_2_	*v*_3_	*v*_4_	*k*	$k_{\Delta /l^{2}}$	$k_{\delta /l^{2}}$		*a*	*c*
LiNO_3_	(1068)	843	1420	738	(10.1)	1.51	(0.727)	0.68	4.692	15.22
NaNO_3_	1068	838	1395	727	10.1	1.50	.683	.94	5.070	16.829
MgCO_3_	(1087)	892	1478	749	(11.2)	1.50	(.646)	.67	4.633	15.015
CoCO3	1090	$\{\begin{array}{c} 838\\ 869 \end{array}$	$\}\begin{array}{c} 1485 \end{array}$	747	11.2	$\{\begin{array}{c} 1.32\\ 1.42 \end{array}$	} .650	.73	4.659	14.957
ZnCO_3_	(1087)	873	1480	745	(11.2)	1.44	(.643)	.74	4.653	15.028
FeCO_3_	(1087)	869	1470	738	(11.2)	1.42	(.623)	.74	4.711	15.436
MnCO_3_	(1087)	870	1480	728	(11.2)	1.43	(.615)	.80	4.777	15.67
CdCO_3_	1075	$\{\begin{array}{c} 837\\ 862 \end{array}$	}1462	724	10.9	$\{\begin{array}{c} 1.32\\ 1.40 \end{array}$	} (.609)	.97	4.930	16.27
MgCa(CO_3_)_2_	(1087)	883	1480	730	(11.2)	1.47	(.618)	.82 (avg)	4.832	15.92
CaCO_3_	1087	881	1432	712	11.2	1.46	.554	.99	4.989	17.062
InBO_3_	(951)	$\{\begin{array}{c} 743\\ 768 \end{array}$	}1288	676	(8.5)	$\{\begin{array}{c} 0.97\\ 1.04 \end{array}$	} (.493)	.81	4.823	15.456
LuBO_3_	(951)	$\{\begin{array}{c} 748\\ 773 \end{array}$	}1275	630	(8.5)	$\{\begin{array}{c} 0.98\\ 1.05 \end{array}$	} (.420)	.85	4.913	16.214

1 Calculated assuming simple valence force potential function for isolated ions.

2 L. H. Ahrens, Geochim. Cosmochim. Acta **2**, 155 (1952).

3 Unit cell data from standard X-ray diffraction powder patterns, NBS circ. 539, vols. **1** to **9**, Dana’s system of mineralogy Vol II, 7th ed; and E. M. Levin and R. Roth in preparation.

**Table 2:** Infrared frequencies and related data for Aragonite structures

Compound	Frequency, cm^−1^				Force constant,^1^ dyne/cm×10^−5^			Ionic radius of cation,^2^ A	Unit cell constants,^3^ A		

	*v*_1_	*v*_2_	*v*_3_	*v*_4_	*k*	$k_{\Delta /l^{2}}$	$k_{\delta /l^{2}}$		*a*	*b*	*c*
KNO_3_	1050	827	1420	714	10.4	1.46	.655	1.33	5.414	9.164	6.431
CaCO_3_	1087	866	$\{\begin{array}{c} 1430\\ 1550 \end{array}$	703 715	}11.2	1.41	$\{\begin{array}{cc} .53 & 0.63\\ .55 & \;.65 \end{array}$	}0.99	4.959	7.968	5.741
SrCO_3_	1074	$\{\begin{array}{c} 845\\ 863 \end{array}$	}1496	701 707	}10.9	$\{\begin{array}{c} 1.35\\ 1.40 \end{array}$	.597 .608	}1.12	5.107	8.414	6.029
PbCO_3_	1053	$\{\begin{array}{c} 826\\ 840 \end{array}$	}1450	670 678	}10.4	$\{\begin{array}{c} 1.29\\ 1.33 \end{array}$	.537 .550	}1.20	5.195	8.436	6.152
BaCO_3_	1060	$\{\begin{array}{c} 845\\ 858 \end{array}$	}1470	695 709	}10.6	$\{\begin{array}{c} 1.35\\ 1.38 \end{array}$	.582 .607	}1.34	5.314	8.904	6.430
NdBO_3_	951	$\{\begin{array}{c} 720\\ 791 \end{array}$	}1307	598 619	}8.52	$\{\begin{array}{c} 0.91\\ 1.10 \end{array}$	.40 .43	}1.04	5.037	7.968	5.741
LaBO_3_	944	$\{\begin{array}{c} 725\\ 790 \end{array}$	}1310	597 613	}8.39	$\{\begin{array}{c} 0.92\\ 1.09 \end{array}$	.40 .42	}1.14	5.104	8.252	5.872

1 Calculated assuming simple valence force potential function for isolated ions.

2 L. H. Ahrens, Geochim. Cosmochim. Acta **2**, 155 (1952).

3 Unit cell data from standard X-ray diffraction powder patterns, NBS Circ. 539 vols. **1** to **9**, Dana’s system of mineralogy Vol II, 7th ed; and E. M. Levin and R. Roth in preparation.

**Table 3:** Infrared frequencies and related data for vaterite structures

Compound	Frequency, cm^−1^				Force constant,^1^ dyne/cm×10^−5^			Ionic radius of cation,^2^ A	Unit cell constants,^3^ A	

	*v*_1_	*v*_2_	*v*_3_	*v*_4_	*k*	$k_{\Delta /l^{2}}$	$k_{\delta /l^{2}}$		*a*	*c*
CaCO	1090	850 878	1450	741 747	9.09	1.46	0.755	0.99	4.12	8.56
				
	Distortion frequencies, *cm*^−1^				Stretching frequencies, *cm*^−1^					
				
LuBO_3_	578, 723				884, 952, 1093			.85	3.725	8.71
YbBO_3_	579, 722				881, 940, 1110			.86	3.732	8.74
TmBO_3_	576, 719				875, 940, 1080			.87	3.748	8.76
ErBO_3_	571, 716				875, 937, 1103			.89	3.761	8.79
HoBO_3_	569, 711				870, 931, 1096			.91	3.776	8.80
DyBO_3_	570, 708				872, 928, 1086			.92	3.791	8.84
YBO_3_	551, 714				874, 935, 1105			.92	3.777	8.81
GdBO_3_	567, 698				862, 922, 1082			.97	3.829	8.89
EuBO_3_	568, 693				860, 922, 1049			.98	3.845	8.94
SmBO_3_	568, 692				851, 910, 1035			1.00	3.858	8.96

1 Calculated assuming simple valence force potential function for isolated ions.

2 L. H. Ahrens, Geochim. Cosmochim. Acta **2**, 155 (1952).

3 Unit cell data from R. W. G. Wykoff, The structure of crystals, 2d ed (1931), and E. M. Levin and R. Roth in preparation.

**Table 4:** Infrared frequencies and related data for miscellaneous structures

Compound	Frequency, cm^−1^				Force constant,^1^ dyne/cm×10^−5^			Ionic radius of cation,^2^ A	Unit cell constants,^3^ A	

	*v*_1_	*v*_2_	*v*_3_	*v*_4_	*k*	$k_{\Delta /l^{2}}$	$k_{\delta /l^{2}}$		*a*	*c*
CsNO_3_	1050	835	1380	716	10.4	1.48	0.623	1.67	10.950	7.716
				
	Distortion frequencies, *cm*^−1^				Stretching frequencies, *cm*^−1^					
				
LaBO_3_	582, 588, 634, 757				952, 1215, 1280			1.14	…………	…………
NdBO_3_	563, 575, 667, 737, 762				935, 1172, 1215. 1310, 1390			1.04	…………	…………
SmBO_3_	561, 571, 674, 732, 760				939, 1055, 1085, 1338, 1388			1.00	…………	…………
EuBO_3_	564, 573, 675, 733, 763				925, 1066, 1180, 1210, 1360			0.98	…………	…………

1 Calculated assuming simple valence force potential function for isolated ion. For details of notation see reference 6 in text.

2 L. H. Ahrens, Geochim. Cosmochim. Acta **2**, 155 (1952).

3 Unit cell data from standard X-ray diffraction powder patterns, NBS Circ. 539, Vol. **9**, p. 25 (Feb. 1960).

## Footnotes

1 Figures in brackets indicate the literature references at the end of this paper.

2 Vaterite was obtained through the courtesy of Dr. Robert Likins of the National Institutes of Health.
